# Supplementary material for: Stacking Model‐Based Classifiers for Dealing With Multiple Sets of Noisy Labels
Source: Biom J. 2025 Mar 12;67(2):e70042. doi: 10.1002/bimj.70042 (PMC11898607; doi:10.1002/bimj.70042)
Supplement: Supplementary file 1 — Supporting Information [file BIMJ-67-e70042-s001.zip › density-based-ensemble-model-main/competitors_real_data/netlab-master/netlab-master/help/gauss.htm]

Netlab Reference Manual gauss


# gauss

## Purpose

Evaluate a Gaussian distribution.

## Synopsis

```
y = gauss(mu, covar, x)
```

## Description

`y = gauss(mu, covar, x)` evaluates a multi-variate Gaussian
density in `d`-dimensions at a set of points given by the rows
of the matrix `x`. The Gaussian density has mean vector `mu`
and covariance matrix `covar`.

## See Also

`gsamp`, `demgauss`

---

**Pages:**
Index


---

Copyright (c) Ian T Nabney (1996-9)
